# Availability of Safe Childbirth Supplies in 284 Facilities in Uttar Pradesh, India

**DOI:** 10.1007/s10995-018-2642-7

**Published:** 2018-11-14

**Authors:** Grace Galvin, Lisa R. Hirschhorn, Maaz Shaikh, Pinki Maji, Megan Marx Delaney, Danielle E. Tuller, Bridget A. Neville, Rebecca Firestone, Atul A. Gawande, Bhala Kodkany, Vishwajeet Kumar, Katherine E. A. Semrau

**Affiliations:** 1 Ariadne Labs | Brigham and Women’s Hospital & Harvard T.H. Chan School of Public Health, 401 Park Drive, 3rd Floor East, Boston, MA 02118 USA; 2 Northwestern Feinberg School of Medicine, Chicago, IL USA; 3 IKS Health, Mumbai, Maharashtra India; 4 Population Services International, Delhi, India; 5 Population Services International, Washington, D.C., USA; 6 Department of Health Policy & Management, Harvard T.H. Chan School of Public Health, Boston, MA USA; 7 Department of Surgery, Brigham & Women’s Hospital, Boston, MA USA; 8 Jawaharlal Nehru Medical College, KLE University, Belgaum, Karnataka India; 9 Community Empowerment Lab, Lucknow, Uttar Pradesh India; 10 Harvard Medical School, Boston, MA USA; 11 Division of Global Health Equity, Brigham & Women’s Hospital, Boston, MA USA

**Keywords:** Childbirth, Quality improvement, Supply, Commodities, Equipment, Essential birth practices

## Abstract

*Objectives* Vital to implementation of the World Health Organization (WHO) Safe Childbirth Checklist (SCC), designed to improve delivery of 28 essential birth practices (EBPs), is the availability of safe birth supplies: 22 EBPs on the SCC require one or more supplies. Mapping availability of these supplies can determine the scope of shortages and need for supply chain strengthening. *Methods* A cross-sectional survey on the availability of functional and/or unexpired supplies was assessed in 284 public-sector facilities in 38 districts in Uttar Pradesh, India. The twenty-three supplies were categorized into three non-mutually exclusive groups: maternal (8), newborn (9), and infection control (6). Proportions and mean number of supplies available were calculated; means were compared across facility types using t-tests and across districts using a one-way ANOVA. Log-linear regression was used to evaluate facility characteristics associated with supply availability. *Results* Across 284 sites, an average of 16.9 (73.5%) of 23 basic childbirth supplies were available: 63.4% of maternal supplies, 79.1% of newborn supplies, and 78.7% of infection control supplies. No facility had all 23 supplies available and only 8.5% had all four medicines assessed. Significant variability was observed by facility type and district. In the linear model, facility type and distance from district hospital were significant predictors of higher supply availability. *Conclusions for Practice* In Uttar Pradesh, more remote sites, and primary and community health centers, were at higher risk of supply shortages. Supply chain management must be improved for facility-based delivery and quality of care initiatives to reduce maternal and neonatal harm.

## Significance

The absence of a single essential supply may mean the difference between life and death for a mother or newborn. Lower level facilities and some districts are severely under supplied in Uttar Pradesh, which may be due to gaps in the supply chain or poor management at the facility or district level. This knowledge will support policy makers and government stakeholders in continuing to play an important role in the efforts to increase facility-based quality of care and improve maternal and newborn outcomes in Uttar Pradesh and globally.

## Background

Despite global progress, maternal and neonatal mortality rates remain high. India has experienced improvements in both rates; however, Uttar Pradesh, India’s most populous state, has death rates well above the country averages. The maternal mortality ratio (MMR) is estimated at 201/100,000 (NITI Aayog 2018a) compared to 130/100,000 nationally; the neonatal mortality rate (NMR) is 40/1000 versus 31/1000 nationally (NITI Aayog 2018b).

Reduction in MMR and NMR is not an easy or straightforward goal: care and experiences in the prenatal, during childbirth, and postnatal periods need to be considered for significant and sustained change. To address health outcomes related to care during childbirth, a popular national strategy has been to encourage facility-based delivery assisted by a skilled provider (Hussein et al. 2004). In 2005, the Government of India implemented the conditional-cash transfer program called Janani Suraksha Yojana (JSY), promoting institutional delivery among women of lower socio-economic status. JSY provides direct cash payment to the mother as well the community health worker that supports care during and post-delivery (Janani Suraksha Yojana (JSY)|National Health Portal of India 2015). JSY has been associated with increased facility-based deliveries, vaccination rates, post-partum checkups, and breastfeeding around the time of delivery (Carvalho et al. 2014). However, the increase in facility-based delivery has not been associated with a significant reduction in MMR and NMR. This may be an indication of poor quality care. An assessment of the quality of care after JSY implementation in a nearby state noted chaotic, unsafe delivery rooms and lack of routine care, including poor techniques, disrespectful and sometimes harmful behavior of staff towards patients (Chaturvedi et al. 2015). Understanding the reasons behind poor quality of care is essential to addressing the problem.

Insufficient availability of supplies and equipment is a major barrier to healthcare workers’ ability to deliver quality care (Tsu 2004). These gaps in supplies and equipment are widely recognized as an important contributor to avoidable maternal and newborn deaths (Kerber et al. 2007; Wagstaff et al. 2004; Zupan 2005) and better equipped facilities were found to provide better quality care (Kruk et al. 2017). The United Nations Commission on Life Saving Commodities for Women and Children estimated that globally, over a 5-year period, 70,000 mothers could be saved by increasing access and delivery of oxytocin and magnesium sulfate. For newborns, increased access and delivery of antibiotics could save 1.2 million babies and correct use of functional resuscitation devices could save 336,000 babies (UN Commission on Life-Saving Commodities for Women and Children 2012). The Government of India (GoI) has made a commitment to make essential medicines available for free to patients (Foy 2012); its Maternal and Newborn Health Toolkit includes the necessary equipment for safe childbirth. However, a survey in 2014 of availability of medicines in Uttar Pradesh’s neighbor state Rajasthan found that availability varies depending on the health setting from 70% at the primary and community health center level to 88% at the district hospital level (Selvaraj et al. 2014b).

In Uttar Pradesh, supplies are allocated through the Central Medical Supply Department (CMSD), a division of the Medical Health Director General who reports to the Principal Secretary for Health and Family Welfare in the State. The CMSD manages the essential drug list and annually negotiates rate contracts with suppliers of consumables and medicines and quantity contracts for items for which the volume is more predictable, such as equipment. In the past, State funds have been used to procure 20% of the medicines from quantity contracts. For the other 80%, districts spend their state-allocated funds through the rate contracts. The Chief Medical Officer (CMO) of the District is in charge of maintaining the stock of supplies for the public health facilities in his or her district. The CMO “pulls” on the state procurement system by requesting supplies from the contracted supplier (quantity contracts) or by using their budget to purchase supplies directly through state-negotiated rate contracts. Allocation of supplies to the facilities in the CMO’s district is based on the requests made by lower level facilities (Selvaraj et al. 2014a).

The World Health Organization (WHO) Safe Childbirth Checklist (SCC) is designed to address gaps in quality of facility-based care through improving the provision of 28 evidence-based, essential birth practices (EBPs) around the time of childbirth. The BetterBirth Program tested the impact of a SCC-based, peer-coaching intervention on maternal and neonatal outcomes in Uttar Pradesh, India (Semrau et al. 2017). During coaching sessions, nurse-coaches identified and worked with frontline staff to overcome barriers to behavior change and utilization of the SCC. One pervasive barrier was a lack of supplies (Hirschhorn et al. 2015; Maisonneuve et al. 2018). Of the 28 practices on the SCC, 22 require supplies, including essential drugs (e.g. oxytocin), consumables (e.g. soap), and equipment (e.g. autoclave, bag and mask). Thus, supply stockouts pose a significant barrier to delivery of high quality maternal and neonatal care. During the BetterBirth trial, supplies were observed as an obstacle to providing care in 10% of coaching sessions (Hirschhorn et al. 2018).

For the BetterBirth Program and other facility-based quality improvement projects, facility data on supplies availability are vital to identify barriers faced by facility staff and identify effective implementation of improvement interventions. Supply data at the facility and district level are also critical for district and state leadership to understand local and system factors associated with supply availability. These data could be used to design and implement interventions to address broader supply system gaps. As part of the site selection process for the BetterBirth trial in Uttar Pradesh, we collected supply availability data in a cross-sectional study (Semrau et al. 2016, 2017). Here, we present information on the pattern of essential birth supplies availability across 284 public health facilities in Uttar Pradesh and associated facility characteristics.

## Methods

### Study Setting

**Fig. 1:**
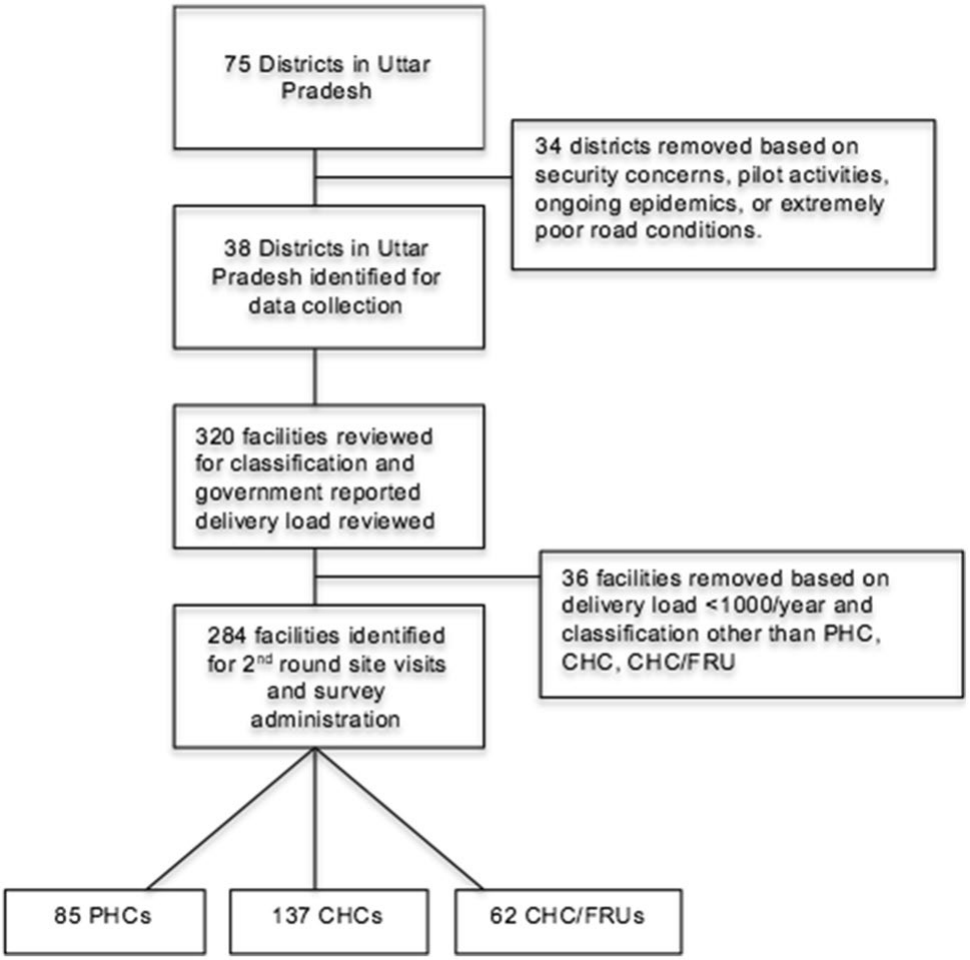
Flow diagram of facilities surveyed for safe birth supplies in Uttar Pradesh, India

In coordination with the Government of Uttar Pradesh and India, 38 of the 74 districts in Uttar Pradesh were selected as potential districts for the BetterBirth trial (Semrau et al. 2017). Districts that had security concerns, health threats from ongoing epidemics, extremely poor road access, or were part of the pilot testing were eliminated. All facilities in the 38 districts (320 facilities) were reviewed for facility classification and delivery load; 284/320 (88.8%) facilities were selected for site visits based on confirmation of facility classification as public-sector primary health centers (PHCs), community health centers (CHCs), or CHC/first referral units (CHC/FRU) and a government reported annual delivery load greater than 1000 in 2012 (Fig. 1). All eligible facilities in eligible districts were surveyed; 2–17 facilities (median 7) were surveyed per district.

Indian Public Health Standards’ guidelines (2012) state that primary health centers (PHCs) are responsible for covering primary health needs of 20,000 to 30,000 people and a target minimum of three deliveries a month (Maternal and Newborn Health Toolkit 2013). As needed, PHC staff refer cases to the community health center level (Indian Public Health Standards: Guidelines for Primary Health Centres 2012). Community health centers (CHCs) cover a population of approximately 80,000 of 120,000 individuals and have 4 PHCs that refer patients to each of them. CHCs are expected to provide a minimum of 10 delivers per month and CHC/FRUs, 20–50 deliveries per month (Maternal and Newborn Health Toolkit 2013). As originally designed, CHCs were planned to provide both basic emergency obstetric and newborn care (EmONC) and cesarean delivery; yet, many CHCs do not function with that capacity (Indian Public Health Standards: Guidelines for Community Health Centres 2012). A CHC/FRU is a facility that has been designated to provide 24-h comprehensive EmONC services: cesarean delivery and blood transfusions (Rural Health Statistics 2014).

### Data Collection

The survey was developed based on the Government of India’s Facility Based Newborn Care Operational Guide published by the Ministry of Health and Family Welfare (2011). The final survey captured information on facility demographics, human resources, maternal and child mortality, and current availability of staff and supplies. These data were planned to be used for matching of facilities in the matched pair, cluster randomized control trial design of the BetterBirth trial. The supplies list was based on the resources needed for the successful implementation of the WHO SCC (Table 1) and represented a subset of the GoI Maternal and Newborn Health Toolkit equipment for labor and delivery plus four drugs listed on the SCC (antibiotics, oxytocin, magnesium sulfate, and Vitamin K). Except Vitamin K, the other three drugs were on the Uttar Pradesh essential drug list (2015).

**Table 1:** Groupings of essentials supplies for safe delivery of normal and uncomplicated birth assessed in 284 facilities in Uttar Pradesh, India

All surveyed supplies (23)	Type of supply	Maternal (8)	Newborn (9)	Infection prevention (6)
Water supply	Consumable			x
Soap	Consumable			x
Gloves	Consumable			x
BP apparatus	Equipment	x		
Oxygen cylinder	Equipment	x		
Mucus extractor/suction machine	Equipment		x	
Ambu bag and mask	Equipment		x	
Delivery tray	Equipment	x		
Labeled newborn corner	Equipment		x	
Towel	Consumable		x	
Cord tie/clamp	Equipment		x	
Any blade (scalpel, knife, blade)	Equipment		x	
Baby weigh scale	Equipment		x	
Any baby warming (bulb or electric)	Equipment		x	
Autoclave	Equipment			x
Boiler	Equipment			x
Chlorine bleach	Consumable			x
IV fluids	Drugs	x		
Magnesium sulfate	Drugs	x		
Oxytocin	Drugs	x		
Antibiotics	Drugs	x		
Vitamin K (injection)	Drugs		x	
Partograph	Equipment	x		

The site visits were performed between July and October 2013 by four teams of two data collectors, including one nurse. Data collectors worked one-on-one with a facility staff member, either facility leader, labor room staff, and/or pharmacist, to locate and inspect supplies. During the assessment, data collectors directly observed and documented supply availability on paper surveys at each facility. Consumables were marked as “available” or “not available”; equipment was marked as “functional”, “not functional”, or “unavailable”; drugs were marked “available” if available and unexpired, or “unavailable” if not present or present but expired (Table 1). If an item was reported as “available” by the facility staff but could not be shown to the data collection team for inspection (e.g. the item was locked up), items were considered “unavailable.” Data were transferred from the paper survey to an online database to collate data across all facilities.

### Ethics and Administrative Approval

This study was approved as a part of the BetterBirth Program, which obtained approvals from the Harvard T.H. Chan School of Public Health Institutional Review Board, World Health Organization Ethics Review Board, Population Services International Research Ethics Board, Community Empowerment Lab Institutional Ethics Board, and Jawaharlal Nehru Medical College-Belgaum ethics review board. The activities were conducted in partnership with the Government of Uttar Pradesh. Permission from the heads of each facility was also requested at each visit; all facilities agreed to participate.

### Data Analysis

To measure general readiness for provision of care of laboring women and their neonates, supplies were grouped into composite measures (Table 1). We grouped supplies with similar function together and only one of the grouped items had to be available to be counted in the composite measure. For example, availability of either knife, blade, or scalpel (collected as separate supplies) was marked as “Yes” for appropriate supply to cut the umbilical cord. Maternal, newborn, and infection control composite measures were categorized and based on the WHO SCC organization.

Supplies availability was calculated as the proportion of total supplies available at each facility. Districts with 6 or more facilities (26 of the 75 districts), were included in the district-level analysis and differences between facilities in each district were calculated using an ANOVA. Mean number of supplies available and differences in availability by facility-type were compared with t-tests.

To measure availability of essential and life-saving drugs, we assessed the percentage of facilities that had availability of all four drugs (antibiotics, oxytocin, magnesium sulfate, and Vitamin K). IV fluids were not included because, alone, IV fluids do not prevent any of the major causes of death during childbirth.

### Regression Analysis

The a priori chosen predictors of supplies availability were: facility type, delivery volume, catchment area population, distance to district hospital, and staff size. Distance to district hospital was chosen as it may impact the ease of supply distribution. Larger catchment area and delivery volume indicate higher turnover and potential strain on the facility. The number of birth attendants could be related to the workload of each staff member and opportunity to track and request supplies when needed.

To ascertain which of these predictors were associated with supply availability, we fit a robust generalized linear model (Wedderburn 1974) with a log link and fixed effects, along with bootstrapped (Efron and Tibshirani 1993) standard errors. This robust approach provides unbiased estimates of the ratio of mean number of supplies available for different covariate values, regardless of the underlying distribution of the number of supplies. Data were analyzed using STATA version 13.1 (College Station, Texas, USA); *p* values < 0.05 were considered statistically significant.

## Results

### Facility Demographics

Of the 284 sites, including 62 CHC/FRUs, 137 CHCs, and 85 PHCs (Fig. 1), mean delivery load was 1998.8 births per year; mean distance to district hospital was 33.1 km; and mean catchment population was 257,100 persons (Table 2). Despite governmental targets, mean delivery load and staff levels did not vary by facility type. As expected, none of the 85 PHCs reported performing a cesarean delivery in the last year, while 3.65% of CHCs and 80% of CHC/FRUs reported at least one cesarean delivery in the last year (Table 2).

**Table 2:** Facility characteristics for 284 public-section labor and delivery facilities across 38 districts in Uttar Pradesh, India

Facility type	N	Catchment population (mean)	Deliveries in 2012 (mean)	Skilled birth attendants (mean)	Distance from district hospital (mean km)	Facilities performing C-sections (%)	Facilities providing blood transfusion (%)
PHC	85	213,044	1908	4.2	31.71	0	0
CHC	137	209,933	2089	4.3	34.72	3.7	< 1
CHC/FRU	62	501,072	1924	4.8	31.61	80	8.1
Total	284	275,100	1998	4.4	33.14	17.3	2.1

### Individual Supplies

**Fig. 2:**
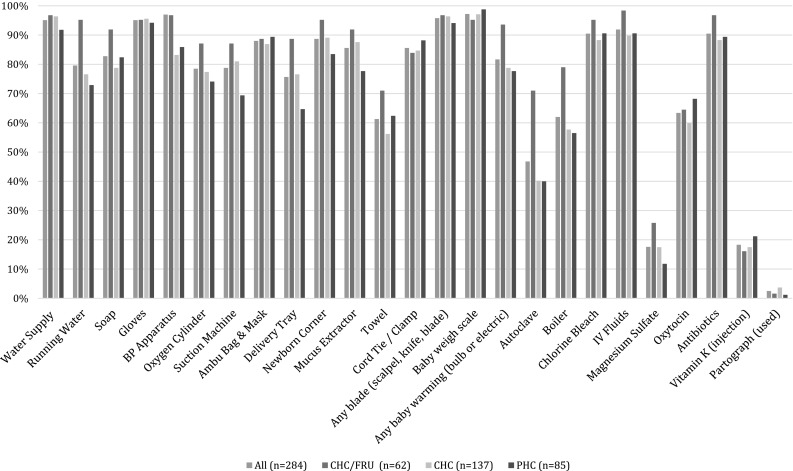
Availability of individual essential supplies for safe delivery, overall and by facility type, in 284 facilities in Uttar Pradesh, India

Of the 23 individual supplies assessed, only six were available in 90% or more of the facilities. The six most widely available supplies were any water supply at the facility for hand washing (running water or otherwise, 95.1%), gloves (95.1%), any blade to cut the cord (95.8%), a baby weigh scale (97.2%), chlorine bleach (90.5%), and antibiotics (90.5%). The following supplies were available in 50% or less of the facilities: functioning autoclave (46.8%), magnesium sulfate (17.6%), and vitamin K (18.3%) (Fig. 2). Of the remaining four essential medicines assessed in the survey, oxytocin was available at 63.4% of facilities and antibiotics were available at 90.5%. Only 24 (8.5%) of the facilities had all four medicines available.

### Cumulative Supplies

None of the 284 facilities had all 23 essential supplies needed to safely deliver women and newborns. On average, facilities had 73.5% (mean number of 16.9, standard deviation (SD): ± 3.27) of supplies available. PHCs and CHCs had similar availability at 71.5% (mean 16.45, SD: ± 3.39) and 72% (mean 16.55, SD: ± 3.16) respectively, with CHC/FRUs having a significantly higher proportion of supplies (79.5%, mean 18.27, SD: ± 2.22, *p* < 0.05) (Table 3).

**Table 3:** Overall and composite supply measures: infection prevention, newborn supplies, and maternal supplies across 284 facilities in Uttar Pradesh, India

Composite measure	TOTAL (*n* = 284)		CHC/FRU (*n* = 62)		CHC (*n* = 137)		PHC (*n* = 85)	
	Mean percentage (%)	Mean (SD) number of supplies	Mean percentage (%)	Mean (SD) number of supplies	Mean percentage (%)	Mean (SD) number of supplies	Mean percentage (%)	Mean (SD) number of supplies
All supplies (23 supplies)	73.5	16.90 (3.13)	79.5	18.27 (2.22)	72.0	16.55 (3.16)	71.5	16.45 (3.39)
Infection prevention (6 supplies)	78.7	4.72 (1.21)	88.2	5.29 (0.95)	76.2	4.57 (1.18)	75.8	4.55 (1.30)
Newborn supplies (11 supplies)	79.1	8.70 (1.68)	84.3	9.27 (1.15)	78.2	8.60 (1.64)	76.7	8.44 (1.97)
Mother supplies (8 supplies)	63.4	5.07 (1.37)	70.0	5.6 (1.03)	62.0	4.96 (1.47)	60.8	4.86 (1.35)

### Maternal, Newborn, and Infection Control Supplies

Availability was also analyzed based on composite measures of supplies: maternal, newborn, and infection prevention (Table 3). Similar to the summary measure of all supplies, CHC/FRUs had significantly more supplies for maternal, newborn, and infection control compared to CHC and PHCs. Of the 8 maternal supplies, the average availability was 63.4% (PHCs 60.8%, CHCs: 62%, CHC/FRUs: 70%). Of the 11 newborn supplies, the average availability was 79.1% (PHCs: 75.2%, CHCs: 78.2%, CHC/FRUs: 84.3%). For infection prevention, the average availability was 78.7% (PHCs: 75.8%, CHCs: 76.2%, CHC/FRUs 88.2%.)

### District-Level Availability

**Fig. 3:**
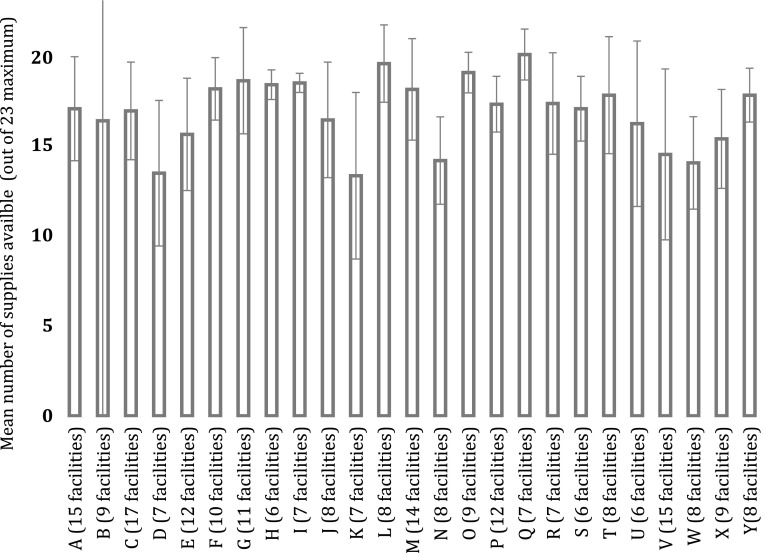
Average availability of supplies for districts with six or more facilities

Because the supply chain depends in part on district-level systems, we assessed supply availability at the district level. Average facility supply availability ranged from 58 to 70% (Fig. 3). Significant differences were seen in average availability between districts (*p* < 0.05).

### Regression Analysis

In fitting the robust generalized log-linear model, only facility type and delivery volume were significant predictors of supply availability. Specifically, facility type CHC/FRU was associated with an 11.1% higher mean supply availability (ratio of means = 1.11; *p* < 0.001) compared with CHC. Distance to district hospital was negatively associated with supply availability (ratio of means = 0.99, *p* = 0.04). As sites were further away from the district hospital, supplies were less available. Delivery volume, catchment area, and number of birth attendants were not correlated with supply availability (Table 4).

**Table 4:** Univariate and multivariable regression analysis: correlation of facility characteristics and supply availability

Characteristic	Univariate models		Multivariable model^a^	
	Ratio of mean number of supplies available Ratio (95% CI)	*p*	Ratio of mean number of supplies available Ratio (95% CI)	*p*
Facility type				
PHC	1.00	–	1.00	–
CHC	1.01 (0.96, 1.06)	0.80	1.02 (0.96, 1.07)	0.560
CHC/FRU	1.11 (1.06, 1.17)	< 0.0001	1.11 (1.05, 1.17)	< 0.0001
Annual delivery volume (*n*)	1.00 (0.99, 1.00)	0.64	–	–
Distance from District Hospital (km)	0.99 (0.98, 1.00)	0.03	0.99 (0.98, 1.00)	0.037
Number of birth attendants (*n*)	1.01 (1.00, 1.03)	0.03	–	–
Catchment area (population)	1.00 (0.99, 1.00)	0.17	–	–

^a^In the multivariate model, using fixed effects, facility type and distance were retained as they were significant at alpha 0.05. Loglinear stepwise regression was used to model the number of supplies (out of 23) available. As the number of supplies is a sum of 23 binary indicators, we used Bootstrap standard errors, resampling by facility to account for correlations among the supplies for a given facility

## Discussion

Lack of availability of essential birth supplies was pervasive across the 284 facilities in Uttar Pradesh and varied by district. In the 284 facilities assessed, none of the facilities had all 23 supplies and only 8% of facilities had all four of the essential drugs assessed. Facility type, district, and distance to the nearest district hospital were significantly correlated with supply availability. These gaps highlight the need to include a systems-based approach to improving critical inputs, such as supplies and staff, in efforts to reduce maternal and neonatal harm.

Not surprisingly, higher-level facilities were more likely to have more supplies overall and across the composite measures. The improved availability of supplies in the CHC/FRUs may be due to focus given to the facilities as they were upgraded from a CHC to a CHC/FRU. In these data, PHCs had a far larger catchment area and higher number of deliveries than initially designed to manage. Here, we saw similar levels of supplies although we expected differences based on delivery load.

The district in which the facility is located was also significantly correlated with supply availability. The centralized system of the CMSD imposes many limitations on the nimbleness of the state and districts to respond to delivery load increases, epidemics, or quality improvement programs. The finding of district-level variability may reflect differences in leadership in that district, including their ability to work within the CMSD restrictions, manage funds, and develop alternate solutions. For example, in a given district, a Chief Medical Officer and his/her staff may have stronger skills and systems for managing supplies through seasonality of birth or unexpected surges in delivery volumes. Other factors influencing district-level supply availability may include proximity and the quality of roads, which is a significant challenge in many parts of India, including Uttar Pradesh (John 2014). Furthermore, closer proximity of a facility to the district hospital was associated with increased supply availability, potentially reflecting ease of getting supplies to the facility from the district hospital.

Across the 284 facilities, delivery volume was not correlated with supplies. This finding was in contrast to results from a study of 124 birth centers in 26 African and 15 Asian countries where low-volume facilities suffered the most severe shortages with only 13 of 23 surveyed supplies available (Spector et al. 2013). The surveyed facilities in this study were not particularly low volume, with averages of 1908 deliveries per year for PHCs and 2089 deliveries per year for CHCs, or an average of 5–six deliveries a day.

The poor availability of the four essential medicines assessed—oxytocin, magnesium sulfate, antibiotics, and Vitamin K—in only 8.5% (24/284) of facilities was extremely concerning, although some of the drugs (antibiotics and oxytocin) were more widely available. Use of oxytocin to augment labor may be an underlying cause of the limited stock (Iyengar et al. 2008; Sharan et al. 2005). A study in Northern Karnataka found similar levels of availability of oxytocin, but higher levels of availability of magnesium sulfate (Jayanna et al. 2016). Potentially, perverse incentives, such as requirement to pay for expired medications, may result in stock shortages. These findings are consistent with other studies from Northern India showing significant gaps in availability of medicines at public health facilities (Prinja et al. 2015). Furthermore, this work is consistent with studies that cite lack of supplies as one of the major reasons for not performing basic obstetric emergency care (Sabde et al. 2016) and that better availability of supplies is correlated with better care (Kruk et al. 2017).

Uttar Pradesh has a procurement system and funds to supply the necessary drugs for each patient. However, the implementation of this system faces challenges: the gaps we have observed, especially in drugs, were significant. Poor maternal and neonatal outcomes in Uttar Pradesh make the timely and appropriate delivery of essential supplies vital to improving quality of care for mothers and their newborns. The Government of Uttar Pradesh along with the World Bank are working to streamline the drug supplies and inventory in the state of UP and have specifically established the Uttar Pradesh Health System Strengthening Project (UPHSSP) to “improve the efficiency, quality, and accountability of health services delivery in Uttar Pradesh by strengthening the State Health Department’s management and systems capacity” (Uttar Pradesh Health System Strengthening Project 2013). Key for this group will be the ability to connect policy with supply availability at the point of care. For example, efforts during the BetterBirth trial to improve supply availability though coaching made modest improvements (Maisonneuve et al. 2018). Yet, more is required to create a procurement system that is reliable and sustainable. Governments must connect policy at the country and state levels and create a supportive and functional system to encourage frontline staff and managers to prevent stockouts.

Our study has a number of limitations. As a cross-sectional study, the data provided a snapshot of supply availability and readiness to utilize the WHO Safe Childbirth Checklist across Uttar Pradesh with supply availability defined as present on the day of the visit. This approach, therefore, did not provide longitudinal data on the frequency and severity of stockouts. Due to decisions to eliminate facilities with extremely poor road access, this study may have underestimated overall availability. Not all supplies needed for the SCC were assessed—including thermometer, stethoscope, fetoscope or Doppler, pads, and HIV test. Thus, we were not able to comment on gaps or availability of these supplies. The assessment tool did not allow for potential substitutes of oxytocin since the survey supply list was based on WHO Safe Childbirth Checklist requirements. For example, misoprostol would not be counted as a substitute for oxytocin; this decision was supported by a recent meta-analysis suggesting that misoprostol is not as effective as oxytocin for prevention of hemorrhage (Mousa et al. 2014).

## Conclusion

Despite high availability of supplies in some facilities, significant room for improvement remains; the absence of a single essential supply may lead to poor outcomes for mothers and newborns. Lower level facilities and some districts are severely undersupplied in Uttar Pradesh, which may be due to gaps in the supply chain or poor management at the facility or district level. Ensuring that leaders at the facility, district and state levels have access to up to date supply information is a critical step to address the supply gaps needed to improve quality of care. UPHSSP has promise to ensure the greater availability of safe birth supplies in UP and should consider the role of facility and district leaders in this initiative and the need for them to be empowered to develop and implement action plans to improve supply availability. Further assessment of the functioning of the supply chain from the facility to district and state level, especially where variability occurs, is needed to identify areas of best practices and where policy or other interventions are needed. This knowledge can support policy makers and government stakeholders in continuing to focus efforts to increase facility-based quality of care and improve maternal and newborn outcomes in Uttar Pradesh and globally.

## Abbreviations

CHC/FRU: Community Health Center/First Referral Unit
CHC: Community Health Center
CMSD: Central Medical Supply Department
EBP: Essential birth practices
EmONC: Emergency obstetric and newborn care
GoI: Government of India
GoUP: Government of Uttar Pradesh
HIV: Human immunodeficiency virus
JSY: Janani Suraksha Yojana
PHC: Primary Health Center
SCC WHO: Safe childbirth checklist
UP: Uttar Pradesh
UPHSSP: Uttar Pradesh Health System Strengthening Project
WHO: World Health Organization
